# Supplementary material for: The immunomodulatory p43 secreted protein of Trichuris whipworm parasites is a lipid carrier that binds signalling lipids and precursors
Source: Sci Rep. 2025 Jul 8;15:24370. doi: 10.1038/s41598-025-08124-w (PMC12238268; doi:10.1038/s41598-025-08124-w)
Supplement: Supplementary file 1 — Supplementary Material 1. [file 41598_2025_8124_MOESM1_ESM.pdf]

The immunomodulatory p43 secreted protein of *Trichuris* whipworm parasites is a lipid carrier that binds signalling lipids and precursors

Malcolm W. Kennedy [1], Allison J. Bancroft [2], Richard K. Grencis [2]

[1] School of Biodiversity, One Health & Veterinary Medicine, Joseph Black Building, University of Glasgow, Glasgow G12 8QQ, Scotland, UK; [2] Lydia Becker Institute for Immunology and Inflammation, Manchester M13 9PT, UK; Manchester Cell Matrix Centre, Manchester M13 9PT, UK; Division of Infection, Immunity and Respiratory Medicine, Manchester M13 9PT, UK. School of Biological Sciences, Faculty of Biology, Medicine and Health, Manchester Academic Health Science Centre, University of Manchester, Manchester M13 9PL, UK.

Correspondence and requests for materials and data should be addressed to malcolm.kennedy@glasgow.ac.uk and richard.grencis@manchester.ac.uk.

Supplementary information

Fig. S1. Hydrophobic ligand binding to the immunomodulatory p43 protein of *T. muris*, and likely irrelevance of an attached dansyl fluorophore.

Fig. S2. *Trichuris muris* p43 internal duplication.

Fig. S3. Comparison of the amino acid compositions of *Trichuris muris* p43 and the orthologue from *Dioctophyme renale* (Dr-DLP-1) against the global average composition of all entries in the SwissProt protein database

Fig. S4. Surface accessibility of internal cavities of p43.

Fig. S5. Alignment of DLP amino acid sequences from three species from the Dorylaimia (Clade I of Nematoda), *Trichuris muris, Trichinella spiralis* and *Dioctophyme renale*.

Fig. S6. The most conserved regions of the three DLPs aligned in Fig. S5 mapped onto p43's structure, and distribution of disulphide crosslinks.

Fig. S1. Hydrophobic ligand binding to the immunomodulatory p43 protein of *T. muris*, and likely irrelevance of an attached dansyl fluorophore. A, subtraction spectrum ((DAUDA + *Trichuris muris* p43) – (DAUDA alone in PBS)) of DAUDA binding to p43 showing the strong blue shift in peak fluorescence emission of the dansyl fluorophore (from 532 nm to 482 nm) when in the apolar environment of the protein's binding site. This shift is identical to that for *T. trichiura's* p47 protein (see main text). **B,** near complete competitive displacement of DAUDA from p43 by oleic acid. **C,** as for **B** but with linoleic acid (like arachidonic acid, a polyunsaturated fatty acid precursor to many biologically active eicosanoid lipids). **D,** binding of dansyl aminocaprylic acid (DACA) to p43 and displacement by oleic acid. DACA bears the same dansyl fluorophore group as does DAUDA, but it is attached by the carboxylate headgroup of the fatty acid instead of at the methyl end of the hydrocarbon tail. This is consistent with the entire fatty acids with their respective attached dansyl group being taken into the binding site. **E, F,** lack of binding of two small dansylated compounds to p43, indicating that the dansyl group itself does not contribute to binding by DAUDA or DACA. **G, H,** meagre displacement of DAUDA bound to p43 by either prostaglandin E_2 or anandamide. Anandamide comprises arachidonic acid (which does displace DAUDA strongly) with an ethanolamide head group. So, a relatively minor change in headgroup can interfere with lipid binding.

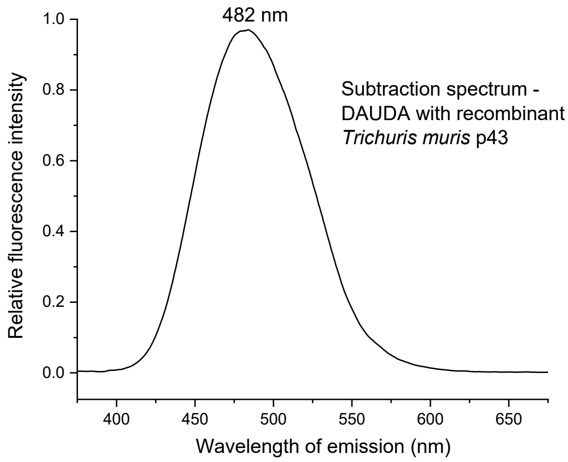

A

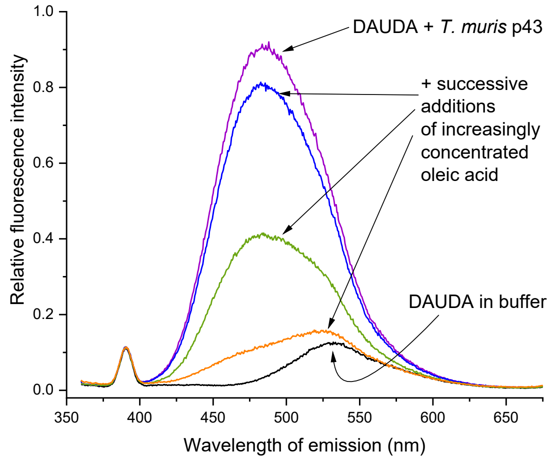

B

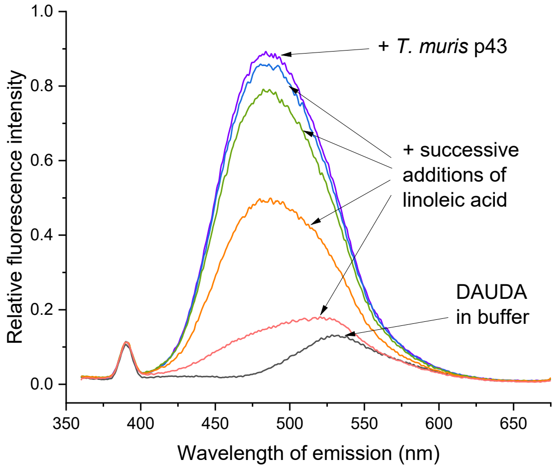

C

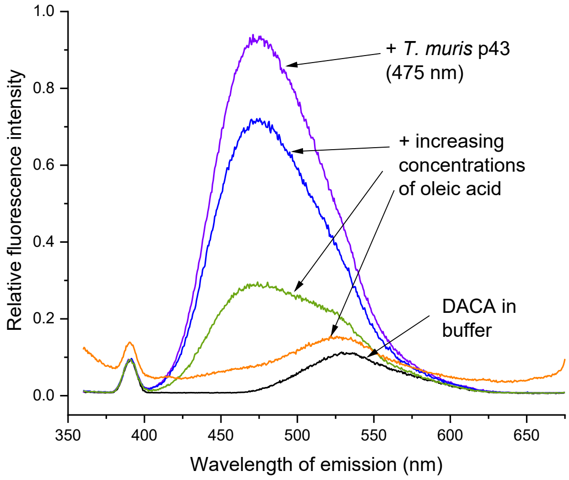

D

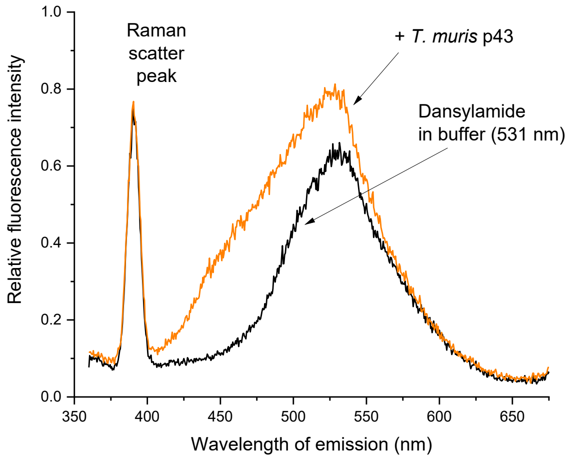
Raman scatter peak
+ T. muris p43
Dansylamide in buffer (531 nm)
Relative fluorescence intensity
Wavelength of emission (nm)

E

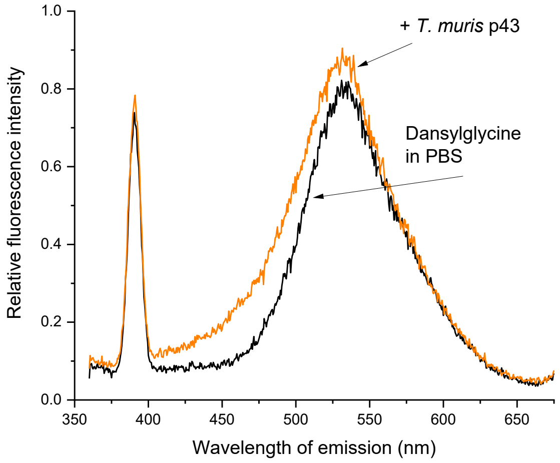
+ T. muris p43
Dansylglycine in PBS
Relative fluorescence intensity
Wavelength of emission (nm)

F

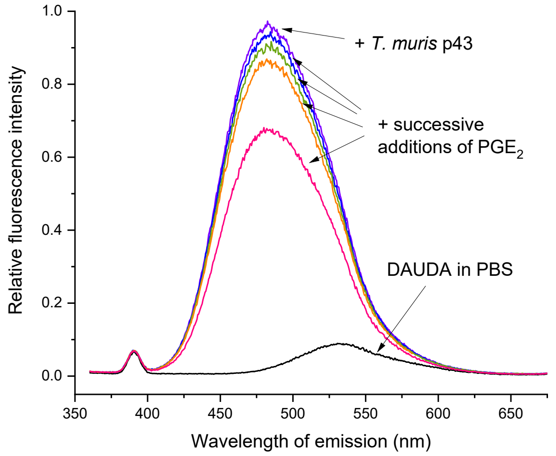
+ T. muris p43
+ successive additions of PGE2
DAUDA in PBS
Relative fluorescence intensity
Wavelength of emission (nm)

G

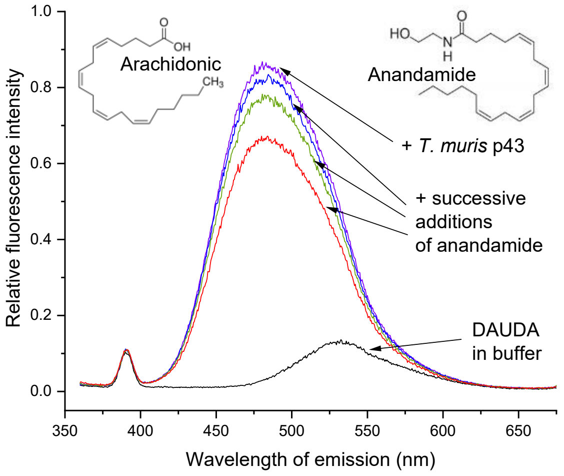
Arachidonic
Anandamide
+ T. muris p43
+ successive additions of anandamide
DAUDA in buffer
Relative fluorescence intensity
Wavelength of emission (nm)

H

Fig. S2. *Trichuris muris* p43 internal duplication. Alignment of N- and COOH- terminal halves of *T. muris* p43. SignalP 6.0 predicts cleavage of secretory signal sequence between position 17 and 18 of the nascent protein. Alignment made with MultAlin, as per Fig. S2.

```
             1        10        20        30        40        50        60        70        80        90        100       110  115
             |--------+---------+---------+---------+---------+---------+---------+---------+---------+---------+---------+----|
Tmp43_Nter   MLVLFFPLLLTVGLSTAGHVKCPDFGDWKPWTDCLWYPPQHMYSKLSHACGMHAHRNLTGVMDL-PHGHKTPPPCGHCSFKFRCRRRPNTEGCYPLDGEVEVCHDHS-----DIC
Tmp43_Cter                    AHDCPAYGPPSEWSECLWFPLKNIVSHVYDHCHVHKEPDGYEPHSVAPANVHIPEKCGFCSFRVKCMKRDKKDGCFPLKLGKKSCGKDDCPTCGDIC
 Consensus   .................ahdCPa%GdpkeWs#CLW%PlqniyShlsdaCgmHaer#gtephdl.PanhhiPekCGhCSFrfrCrrRdnk#GC%PLdgekesCgddd.....DIC

             116      125       135       145       155       165       175       185       195       205       215       225228
             |--------+---------+---------+---------+---------+---------+---------+---------+---------+---------+---------+--|
Tmp43_Nter   TLPKLPHLGCGYAFINEKLKQCFTRPDT----PSYVRLGYRKMFESIPKKHCIEKDGMCKCCCGDYEPNESGTECIKPP
Tmp43_Cter   TLDKINGSCAFPRVMKEKIWDDFTATSKEKHMPHWKRDGYAKMLMQLPYSNCKEVGDKCKCCCHPYEPNKDGTACVVKEYCKRVHELHHHDHHGHGEEHHKSSSSESKEHHHH
 Consensus   TLdKinglcagpafinEKik#dFTapdk....PhwkRdGYaKMleqiPkknCiEkddkCKCCCgdYEPNedGTaC!kke.................................
```

Fig. S3. Comparison of the amino acid compositions of *Trichuris muris* p43 and the orthologue from *Dioctophyme renale* (Dr-DLP-1) against the global average composition of all entries in the SwissProt protein database (https://web.expasy.org/docs/relnotes/relstat.html). **A.** *T. muris* p43, cleavable secretory signal sequence as predicted by SignalP removed, so count began from position 18 at amino acids GHV in the sequence; count truncated just before the histidine-rich tail at position HEL so as properly to compare the main sequences of the two proteins. **B,** The same analysis for Dr-DLP-1/P44 of *D. renale,* which does not have a long histidine-rich COOH-terminal tail. This illustrates the near-identical enrichment with cysteines in both proteins and above average histidine richness typical of this family of proteins even excluding any histidine-rich tails (such as in in *Trichuris* and *Trichinella* species *spp.* and *Trichinella spp.*) although the level of His-richness of Dr-DLP-1 is less apparent. Linear curve fits; intercept set for zero; 95% confidence bands coloured. The unusual proline richness in both proteins may relate to maintaining crucial structural elements, though again, p43 shows this more than does Dr-DLP-1. *T, muris* p43 sequence from Genbank NCBI 6QIX_A; *D. renale* Dr-DLP-1 sequence from GenBank NCBI QTE33903.2

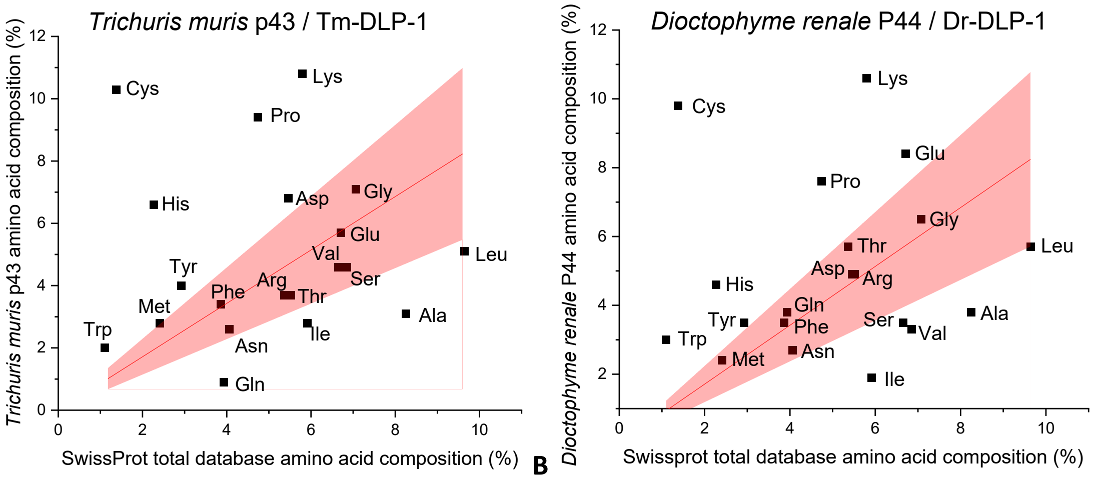

A **B**

Fig. S4. The internal cavities of p43 are surface-accessible. Surface accessibility of internal cavities of p43. X-ray crystal structure of p43 (PDB 6QIX_A) showing the two molecules of the asymmetric unit with the components of the crystallisation buffer *in situ* (magenta sticks), some of which are on the surface of the protein, but many are internal.

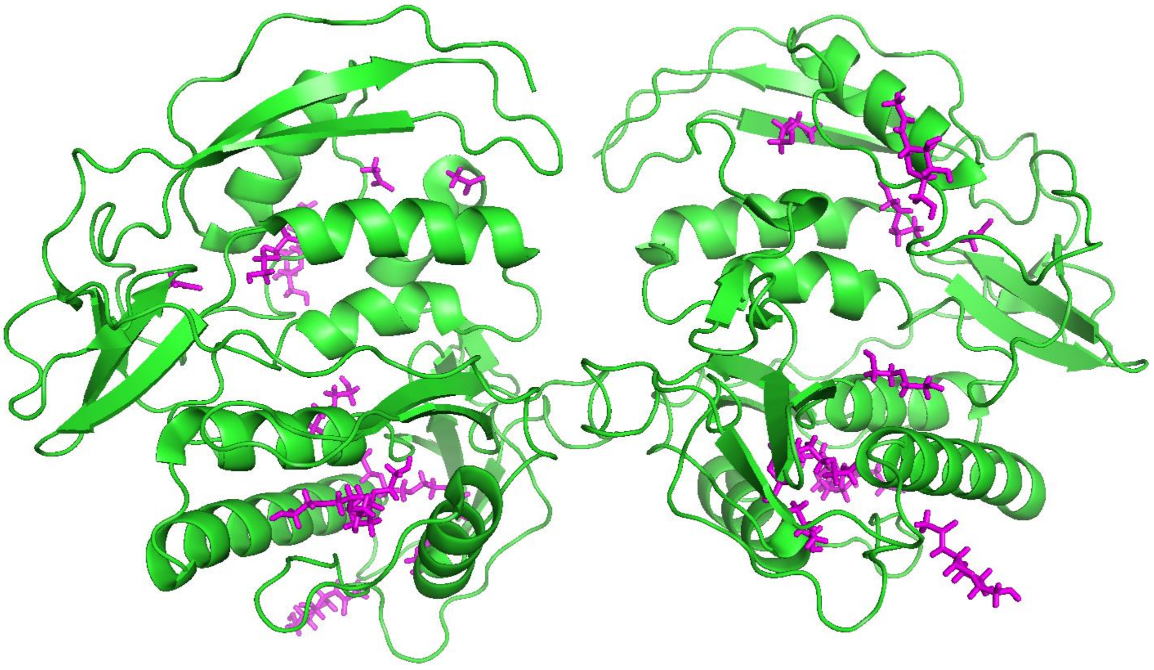

Fig. S5. Alignment of DLP amino acid sequences from three species from the Dorylaimia (Clade I of Nematoda), *Trichuris muris, Trichinella spiralis* and *Dioctophyme renale*. Also a phylogram from these and other DLP sequences that are not shown - note that the *Soboliphyme baturini* sequence is currently incomplete and not included in the alignment. Multiple alignment and phylogram made with MultAlin set for the Blossum62 matrix. In the consensus line lower case blue font represents amino acids that are identical in two out of the three protein positions, and to illustrate amino acid similarities ! is anyone of IV; $ is anyone of LM; % is anyone of FY; # is anyone of NDQEBZ. The *T. spiralis* sequence was obtained from NCBI accession KAL1244686.1, the *T. muris* from NCBI 6QIX_A; the *D. renale* from NCBI QTE33903.2.

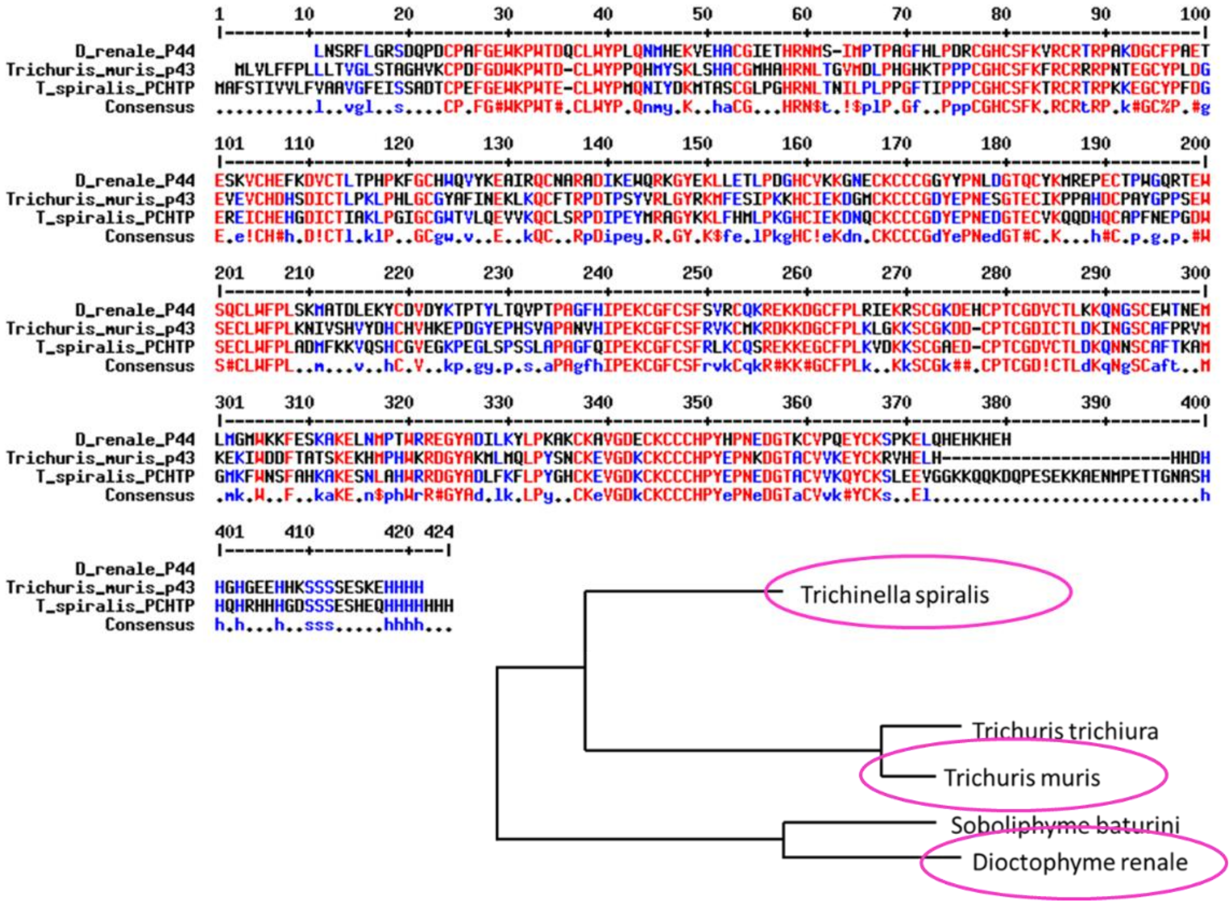

Fig. S6. The most conserved regions of the three DLPs aligned in Fig. S5 mapped onto p43's structure, and distribution of disulphide crosslinks. A. Two orientations of the p43 x-ray crystal structure in which the most conserved regions are coloured magenta as delineated from the amino acids sequence alignments of DLPs from three species of Clade I nematode in Fig. S5. These regions were chosen following the rule that each region must have at least four consecutive amino acids that are either identical or biochemically similar (coloured red in the consensus line, and similarity as defined in the legend to Fig. S5), and extended in either direction until interrupted by more than two non-identical or biochemically dissimilar amino acids. This shows that the most conserved regions are the extended/β structures and unstructured regions on or near the surface of the protein, potentially therefore involved in interactions with other structures within the nematodes, such as other proteins and membranes, in performing conserved physiological functions. **B.** Two molecules of p43 as in the x-ray crystal asymmetric unit in which the Cys-Cys disulphide cross links are coloured magenta, showing their relative concentration in the more unstructured external loop regions.

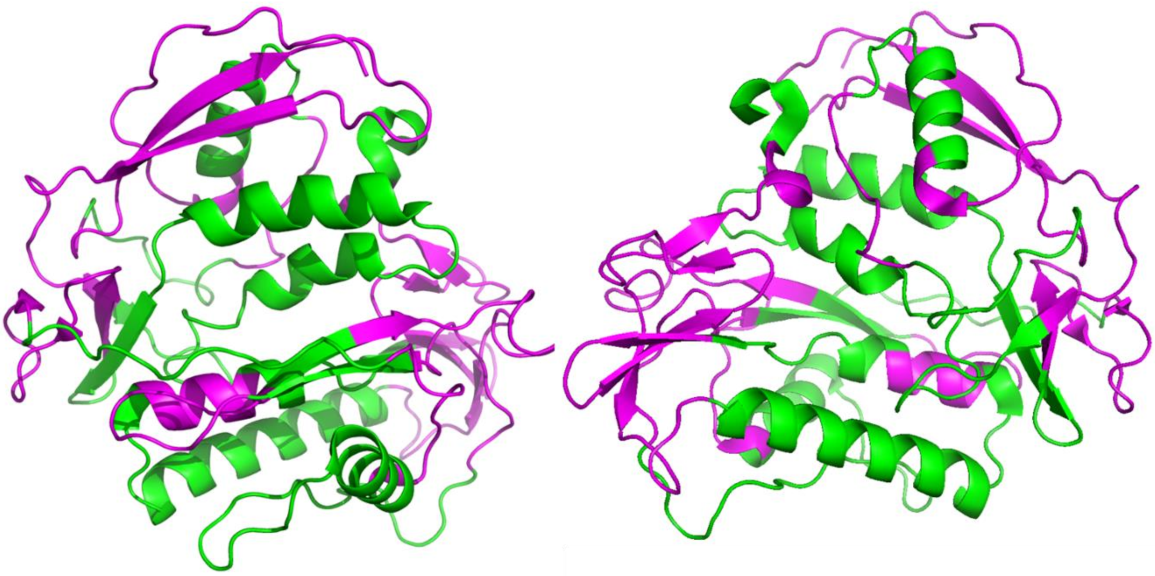

A

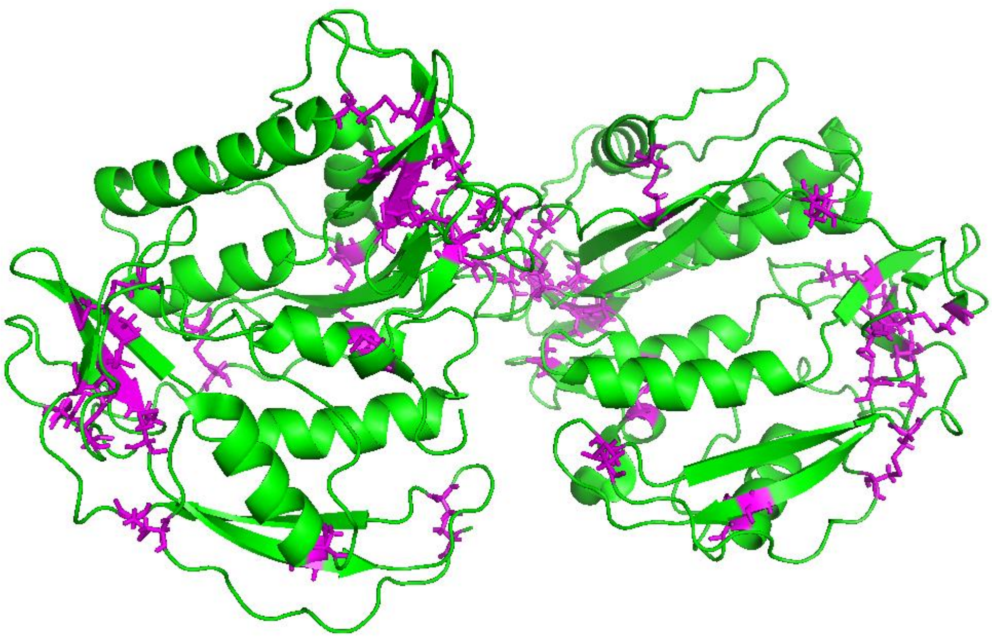

B
